# Celastrol Improves Preference for a Fatty Acid, and Taste Bud and Systemic Inflammation in Diet-Induced Obese Mice

**DOI:** 10.3390/nu17081308

**Published:** 2025-04-09

**Authors:** Manal Benmouna, Chahid Benammar, Amira Sayed Khan, Fatima Zohra Djeziri, Aziz Hichami, Naim A. Khan

**Affiliations:** 1 Physiologie de Nutrition & Toxicology (NUTox), UMR UB/INSERM 1231 Center for Cellular & Translational Molecular Medicine (CTM), Université Bourgogne Europe, & FCS Bourgogne-Franche Comté, LipSTIC LabEx, 21000 Dijon, France; benmanel84@gmail.com (M.B.); chabena62@yahoo.fr (C.B.); fatimazohradj@yahoo.fr (F.Z.D.); 2 Laboratoire des Produits Naturels (LAPRONA), Université Abou Bekr Belkaid, Tlemcen 13000, Algeria; amira.khan@u-bourgogne.fr (A.S.K.); aziz.hichami@u-bourgogne.fr (A.H.)

**Keywords:** fat taste, terpenoid, gustation, CD36, GPR120, inflammation, liver, high-fat diet

## Abstract

Background: Obesity is associated with the altered gustatory perception of dietary fatty acids. Celastrol, a triterpene, has been demonstrated to exert anti-obesity effects in rodents. We assessed the role of Celastrol in the modulation of the oro-sensory perception of lipids in control and high-fat diet (HFD)-induced obese mice. Methods: Male mice of the C57B/6J strain were fed a HFD for 11 weeks and then were administered or not with Celastrol further for 4 weeks. The body weight was recorded weekly. Before the sacrifice, the animals were subjected to oro-sensory detection of a dietary long-chain fatty acid in a two-bottle choice paradigm. After the sacrifice, the fungiform taste buds were isolated and analyzed for mRNA expression, encoding fat sensors (CD36 and GPR120) and pro-inflammatory cytokines (IL-1β, IL-6 and TNF-α). Circulating concentrations of IL-6 and TNF-α were also determined, and liver was used to analyze the mRNA expression of lipogenic genes. Results: Celastrol administration in obese mice decreased body weight and also re-established the loss of oro-sensory perception for a dietary fatty acid, and this phenomenon was, in part, due to the upregulation of mRNA, encoding fat taste receptors (CD36 and GPR120) in tongue taste bud cells. Furthermore, Celastrol decreased inflammation both in taste buds and blood circulation. Conclusions: Our findings suggest that Celastrol decreases body weight gain, ameliorates the gustatory perception of lipids, and downregulates inflammation in obese mice.

## 1. Introduction

Obesity, a global epidemic, is characterized by the excessive accumulation of fat in adipose tissue. According to the WHO [1], there are more than 1.9 billion adults who are overweight, and more than 777 million adults who are clinically obese. Overweight and obesity represent the fifth leading cause of death worldwide. Each year, 2.8 million adults die from these conditions: 44% because of diabetes, 23% by ischemic heart disease, and 7 to 47% of deaths are caused by certain cancers that are also attributed to overweight and obesity [1].

Obesity is a multifactorial disease, including genetic susceptibility under the influence of environmental factors and other factors such as diet, excessive sedentary lifestyle, and a lack of physical activity. The interactions between these factors may create a favorable pattern for the expression of genes that may trigger obesity [2]. It is generally accepted that eating fat-rich food may contribute to increased fat accumulation and increases the incidence of obesity. In addition, several studies have reported a relationship between fat accumulation and chronic inflammation that promote insulin resistance in the obese. In obesity, the pro-inflammatory mediators released by inflamed adipose tissue target the liver and, consequently, trigger hepatic steatosis, which is considered the initial stage of non-alcoholic steatohepatitis [3]. The inflammatory condition in the liver of the obese is so important that, even after weight loss, signs of inflammation, like fibrosis, in the liver, still persist and may contribute to an increased risk for rebound weight gain [4]. We, therefore, in the present study, assessed the inflammatory cytokines in the liver of obese animals before and after Celastrol administration.

Obesity is also associated with a strong attraction for dietary fat in rodents [5]. In human beings, several studies have shown that obese subjects prefer lipids compared with lean subjects [5,6]. There is a positive correlation between obesity and fat detection thresholds; i.e., the higher the body mass index (BMI), the higher the oro-sensory detection threshold in obese participants [7]. It is important to mention that a fat-rich obesogenic diet that leads to obesity also alters fat taste perception, viz., increasing fat intake causes a decrease in fat taste detection sensitivity. Multiple cross-sectional studies, conducted on human volunteers, have confirmed these observations [8,9], meaning that individuals who consume high fat-rich food were found to have reduced taste oral sensitivity to fat. One of the plausible mechanisms involved in the attraction to dietary lipids is the attenuated function of CD36, expressed by tongue taste bud cells. Indeed, CD36 acts as a sensor to detect and to respond to dietary fat during mastication to signal to the brain and help regulate fat intake [10,11]. Several studies on CD36 SNP have demonstrated reduced sensitivity (or high threshold) to the taste of fat that may lead to fat overeating or a high preference for fat-rich foods in the obese [7,12]. Hence, an anti-obesity strategy should trigger the restoration of reduced fat taste sensitivity.

Over the past two decades, chemical substances derived from plants have attracted public and scientific interest in their role in preventing disease and maintaining good health [13]. Several epidemiological studies suggest that plant-based foods rich in polyphenolic agents reduce inflammation [14]. In vivo and in vitro studies suggest that pentacyclic triterpenes, purified from plants, modulate different factors linked to metabolic syndrome [14]. These phytochemicals could be promising candidates for clinical trials for the treatment of metabolic syndrome. They can be powerful anti-obesity agents because they regulate the different stages of adipogenesis, lipolysis, and fatty acid oxidation and target transcription factors involved in adipocyte development [15]. Our team proposes that certain molecules of the terpenoid family can play a role in the modulation of gustatory perception of fat and exert beneficial effects in obesity in the mouse [16,17]. Oleanolic acid, a triterpene purified from *Olea europaea*, exerts an anti-obesity effect in mice [18]. Zizyphine, a triterpene purified from *Zizyphus lotus*, and oleanolic acid exhibit close structural homology to taurolithocholic acid (TLC), a bile acid, which acts via TGR5 (Tekada-G-protein-receptor-5, a bile salt receptor). Indeed, our team has identified the expression of TGR5 in mice and human taste bud cells [19].

Celastrol is a naturally occurring triterpenoid isolated from Celastraceae plants such as *Tripterygium wilfordii* [20] and *Celastrus orbiculatus* [21]. It is noteworthy that these two plants have been used in Chinese medicine to treat inflammatory pathologies like rheumatoid arthritis [22]. Celastrol has been purified as an active ingredient from these plants [23]. Since the anti-obesity effect of Celastrol has been reported [24], and fat intake is altered in obesity as mentioned here before, we undertook the present study to assess the effects of Celastrol on the modulation of fat preference and inflammation in diet-induced obesity in mice.

## 2. Materials and Methods

### 2.1. Materials

Celastrol was purchased from Ficher Scientific (Illkirch-Graffenstaden, Strasbourg, France), and the standard diet was purchased from SAFE (Route de Saint Bris, Augy, France). The palm oil was purchased from Huilerie Vigean (Clion, France). All of the solvents and other products were obtained from Merck (St. Quentin Fallavier, Lyon, France). The ELISA kits for TNF-α (ref. LS-F12798-1) and IL-6 (ref. OKBB00190) were purchased from Clinisciences (Nanterre, France).

### 2.2. Animals and Diets

Eight-week-old male C57B/6J mice were obtained from Janvier Elevage (Le Genest-St-Isle, France). The general guidelines for the care and use of laboratory animals, recommended by the council of European Economic Communities, were followed. The experimental protocol (C21231008EA) was approved on 05/08/2021 by the Regional Ethical Committee of Burgundy (France). Mice were housed in animal husbandry facility under controlled conditions at a constant temperature (20 °C ± 2) and humidity (60 ± 5%), with light/dark cycle of 12 h with food and water ad libitum. The mice (n = 15) were divided into two groups: standard (Std) diet-fed group (n = 5) and HFD-fed group (n = 10). The palm oil was the main fat component in high-fat diet. The different diets and their fatty acid compositions can be seen in Table 1 and Table 2. The diets were prepared every week and stored at 4 °C until further use.

### 2.3. Diet-Induced Obesity

C57B/6J male mice were fed a high-fat diet (HFD) for 15 weeks. After 11 weeks of HFD, obese animals were divided into two groups: one continued to receive HFD and vehicle, whereas another received the same HFD and Celastrol at 100 µg/kg/day [25] intraperitoneally for four weeks further. Mice were weighed weekly, and food and energy intake were determined daily. After 15 weeks, mice were fasted overnight and sacrificed by using isoflurane to anesthetize. Serum was isolated by centrifugation from clotted blood, and liver samples were weighed and immediately stored at −80 °C until analysis. Tongues were immediately removed and placed in Tyrode solution for papillae isolation.

### 2.4. Determination of Pro-Inflammatory Cytokines

At the time of sacrifice, blood was drawn in dry tubes and centrifuged at 200× *g* × 10 min. The supernatant/serum was stored at −20 °C until the dosage of cytokines (IL-6 and TNF-α). The ELISA kits were used for the quantification of IL-6 and TNF-α, and the dosages were performed as per manufacturer’s protocol, furnished with the kits.

### 2.5. Liver Cholesterol and Triglyceride Determinations

Liver samples were thawed and homogenized in 9 mL of chloroform–methanol (2:1) solution. Methanol (3 mL) was added into the homogenates that were vortexed and further centrifuged (3000× *g* × 15 min). The resulting supernatant (8.25 mL) was transferred to glass tubes and was added with 4 mL of chloroform and 2.75 mL of 0.73% NaCl, and it was further centrifuged (3000× *g* × 3 min). The lower phase, after evaporation, was resuspended in 1 mL of buffer containing 1,4-piperazinediethanesulfonic acid (28.75 mM), magnesium chloride (57.76 mM), free fatty acid–bovine serum albumin (8.76 microM), and sodium dodecyl sulfate (0.1%), and lipids were emulsified by sonication [25]. Total cholesterol and triglyceride (TG) levels were determined by colorimetric enzymatic methods (DiaSys, Holzheim, Germany).

### 2.6. Two-Bottle Preference Test

In order to study the preference for a lipid solution, we employed a two-bottle preference test, according to previously published procedure [17]. Before starting the experiment, mice were provided with two drinking bottles for 24 h. The mice were further subjected to two bottles: one contained 0.1% of linoleic acid in gum of xanthan, GX (0.3%, *w*/*v*) in water, and the other bottle contained vehicle, GX (0.3% *w*/*v*). The intake was determined by weighing the bottles after 12 h, i.e., overnight.

### 2.7. Isolation of Mouse Taste Bud Cells

Tongue fungiform papillae were dissected under microscope. The mouse taste bud cells (mTBCs) were isolated, as previously described [18], by enzymatic dissociation by using the mixture of elastase and dispase, 2 mg/mL each in Tyrode buffer: 120 mM of NaCl, 5 mM of KCl, 10 mM HEPES, 1 mM of CaCl_2_, 10 mM of glucose, 1 mM of MgCl_2_, 10 mM of Na pyruvate, and pH 7.4. Isolated mTBCs were stored at −80 °C for RT-qPCR analysis.

### 2.8. mRNA Expression by Real-Time-Quantitative PCR (RT-qPCR)

The sequences of the primers used can be seen in Table 3. Total RNA was extracted from liver and taste bud cells by using TRizol (Fischer Scientific, Illkirch-Graffenstaden, Strasbourg, France), then treated with DNAase, and reverse-transcribed with cDNA synthesis kit according to the manufacturer’s instruction.

RT-qPCR was performed using stepOnePlus real-time PCR system by employing SYBR Green I (Merck, St. Quentin Fallavier, Lyon, France). The relative gene expression was determined using (∆Ct) by the comparative 2⁻CT method. Beta-actin was used as a housekeeping gene.

### 2.9. Statistical Analysis of Data

Results are expressed as mean ± SD (n = 5). The significance of difference between mean values was determined by analysis of variance (ANOVA), followed by Tukey’s least-significant-difference (LSD) test. Significant differences were considered at *p* < 0.05.

## 3. Results

### 3.1. Celastrol Decreases Body Weight Gain and Modulates Fat Preference in HFD-Fed Mice

**Figure 1:**
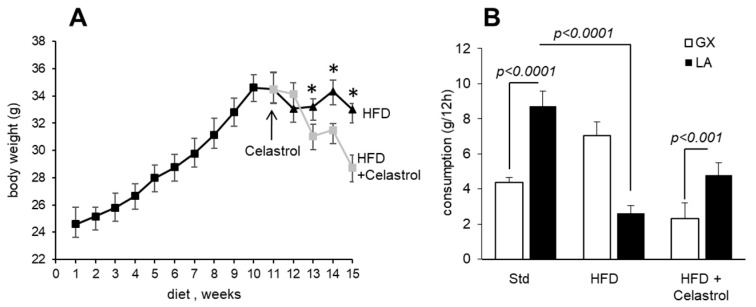
Effect of Celastrol on diet-induced obesity and preference for fat: (**A**) Mice maintained on a HFD for 15 weeks (n = 10). On the 11th week of a HFD, obese animals (n = 10) are divided into two groups: one is continued on a HFD alone (filled triangles), whereas the second one is fed the same HFD and administered with Celastrol (100 µg/kg/day) intraperitoneally for 4 more weeks (gray squares). The arrow in the Figure shows the 11th week of the HFD when the mice are divided into two groups. The body weight is measured weekly. The asterisks (*) show significant differences between two groups (*p* < 0.01). (**B**) Another group of mice (n = 5), maintained on a standard diet for the same duration, i.e., 15 weeks, is used as the control group from Figure 1B, Figure 2, Figure 3 and Figure 4. After 15 weeks, the animals are deprived of water for 6 h, and a two-bottle preference test is performed. GX, gum of xanthan; LA, linoleic acid.

Figure 1 shows the body weight gain in mice maintained on a HFD during 15 weeks. Mice maintained on a HFD gained weight as a function of time (Figure 1A). However, the body weight remained almost the same from the 11th week onwards in HFD-fed animals. Interestingly, a significant and progressive reduction in body weight gain was observed after 4 weeks of Celastrol administration in obese animals.

In order to compare gustatory attraction for lipids in mice, the two-bottle preference test was performed using a dietary long-chain fatty acid (LCFA), i.e., linoleic acid, 0.1% (*w*/*v*). Hence, the individually caged mice were allowed to choose between 0.1% LCFA emulsified in 0.3% xanthan gum in water or water with vehicle alone (0.3% xanthan gum) over a period of 12 h. The standard diet-fed mice showed a strong preference for a solution containing LCFA; however, HFD-fed mice showed significantly decreased preference for this fatty acid. Interestingly, Celastrol treatment upregulated the preference for LCFA in mice maintained on a HFD (Figure 1B).

### 3.2. Celastrol Modulates Fat Taste Receptor and Pro-Inflammatory Cytokines mRNA Expression in HFD-Fed Mice

**Figure 2:**
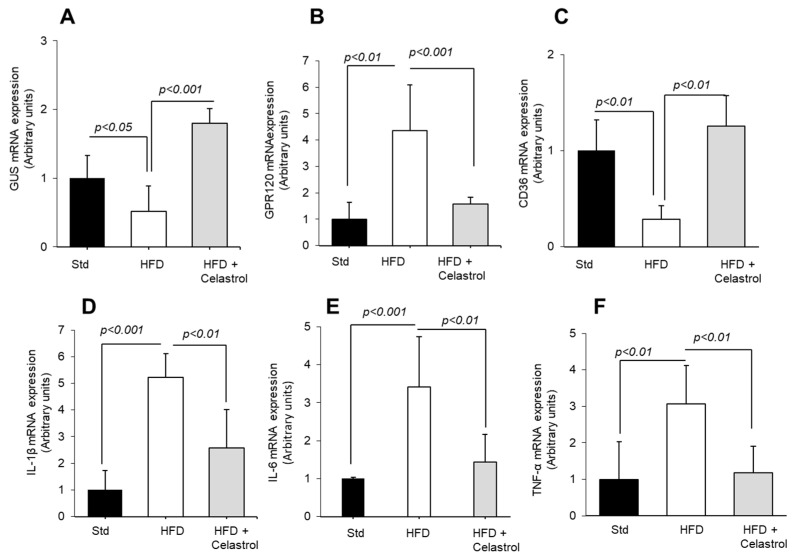
Effect of Celastrol on the expression of mRNA encoding gustducin, fat taste receptors, and pro-inflammatory cytokines in taste bud cells. After 15 weeks of the experiments (see Figure 1 legends), the animals are sacrificed, and fungiform taste papillae are recuperated to isolate taste bud cells that are subjected to RT-qPCR analyses for gustducin, GUS (**A**), GPR120 (**B**), CD36 (**C**), and pro-inflammatory cytokines, IL-1β, IL-6, and TNF-α (**D**–**F**) mRNA expression in mTBCs.

To explore whether the alteration in fatty acid preference in obese mice is due to the altered expression of fat taste receptors in tongue epithelium, we examined the mRNA expression of main lipid receptors (CD36 and GPR120) and also gustducin (GUS), a marker of taste receptor cells, in mouse taste bud cells (Figure 2A–C). We observed that there was a significant decrease in taste bud CD36 and GUS mRNA expression in HFD-fed mice. Obese mice that received Celastrol exhibited significantly higher CD36 and gustducin mRNA expression than HFD-fed mice. However, GPR120 mRNA did not follow the same trend, its expression was upregulated in obese mice, and Celastrol curtailed the same.

We further examined the mRNA levels of pro-inflammatory cytokines (IL-1β, IL-6, and TNF-α) in mTBCs isolated from obese mice administered or not with Celastrol. As shown, the mRNA expression of genes, like IL-1β, IL-6, and TNFα, was increased by the HFD, and their expression was downregulated by Celastrol treatment (Figure 2D–F). In this study, it would have been better, if we had determined the expression of lipid sensors at the protein level by Western blot.

### 3.3. Celastrol Decreases the Circulating Concentrations of Pro-Inflammatory Cytokines

**Figure 3:**
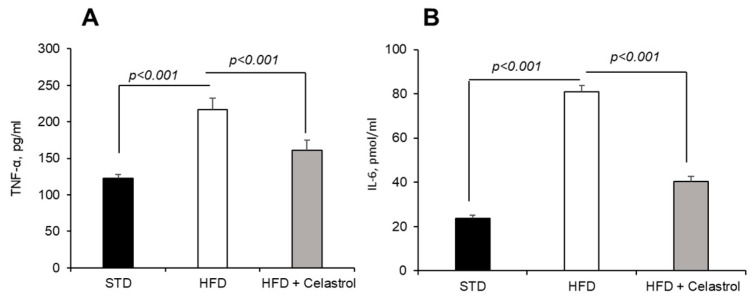
Effect of Celastrol on circulating pro-inflammatory cytokines in mice. The protocol is identical as described in Figure 1 and mentioned in Section 2. The figure shows the levels of pro-inflammatory cytokines, TNF-α (**A**) and IL-6 (**B**).

Since obesity is associated with low-grade inflammation, we determined blood concentrations of IL-6 and TNF-α by ELISA (Figure 3A,B). The concentrations of IL-6 and TNF-α increased in HFD-fed mice, whereas Celastrol lowered their levels in obese mice (Figure 3A,B).

### 3.4. Celastrol Regulates Hepatic Lipid Levels and mRNA Expression of Lipid Metabolic Mediators

**Figure 4:**
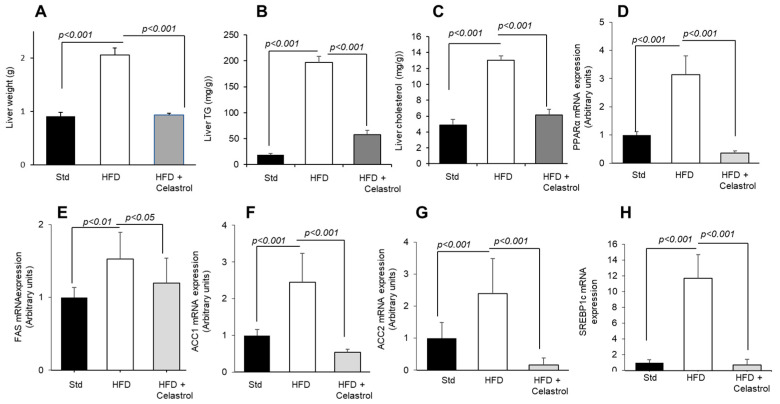
Effect of Celastrol on the expression of hepatic mRNA encoding lipogenic genes. The protocol is identical as described in Figure 1 and mentioned in Materials and Methods. After 15 weeks of experimentation, the animals are sacrificed, liver was weighed (**A**), and liver TG (**B**) and cholesterol (**C**) concentrations were determined. The liver was also used for the mRNA expression of metabolic genes, i.e., PPARα (**D**), FAS (**E**), ACC1 (**F**), ACC2 (**G**), and SREBP1c (**H**).

At first, we determined liver weight, and we observed that feeding a high-fat diet increased liver weight, and Celastrol treatment decreased the same significantly (Figure 4A). Interestingly, there was an increase in triglycerides and cholesterol levels in the liver of animals, maintained on a high-fat diet, and Celastrol decreased their liver concentrations (Figure 4B,C).

We also examined the genes involved in lipid metabolism. Several findings suggest that the activation of PPARα is involved not only in lipid metabolism but also in the induction of “acute phase response” of inflammation in the liver [26,27,28]. Consequently, PPARα gene deficiency has been found to downregulate the mRNA of certain pro-inflammatory agents, like TNF-α, IL-1β, and IL-6 in the mice [29]. Our results suggest that the anti-inflammatory action of Celastrol in the liver might be, in part, contributed by its action on lowing PPARα mRNA expression (Figure 4D).

The ACC and FAS have been considered as hepatic biomarkers of lipogenesis and the de novo synthesis of fatty acids [26]. FAS is principally involved in fat storage when energy-dense food is consumed. Concerning ACC, there are two isoforms: ACC1 has been identified in cytosol and involved in the rate-controlling reaction of de novo lipogenesis, whilst ACC2 is embedded into the mitochondria plasma membrane and regulates fatty acid oxidation by producing malonyl-CoA [27]. The inhibitory action of Celastrol on FAS and ACC (1 and 2) mRNA expression (Figure 4E–G) in HFD-fed mice shows that this terpenoid decreases lipogenesis, triggered by the high-fat diet and, consequently, may result into low fat mass in obese mice during high-fat diet feeding. Furthermore, the SREBP1c, a transcription factor, which regulates lipid synthesis in the liver, was also upregulated in the liver of obese mice, and Celastrol further decreased the expression of its mRNA in liver (Figure 4H).

## 4. Discussion

Celastrol, a bioactive compound, purified from medicinal plants like *Tripterygium wilfordii*, is a herb used in Chinese medicine and has been shown to exert anti-obesity effects [24,30]. In the present study, we extended these observations on other aspects of obesity, for example, the oro-sensory perception of a long-chain fatty acid (LCFA), the expression of fat taste receptors, liver lipogenesis, and inflammation. Celastrol treatment reduced body weight in obese mice in accordance with the report of Liu et al. [24] who demonstrated its anti-obesity action via increasing leptin sensitivity to hypothalamus and improving insulin sensitivity by inhibiting the NF-κB pathway. It is also possible that, in addition to leptin sensitivity, Celastrol decreased body weight by increasing energy expenditure and improving gut microbiota as reported in a rat model [31].

Nutritional obesity is generally associated with high-fat, rich food intake. It has been reported that diet-induced obesity in mice is associated with the low oro-gustatory detection of long-chain fatty acids, and this phenomenon might contribute to high fat intake [10]. Similarly, in several studies on human populations, the CD36 genetic polymorphism, responsible for the attenuated function of CD36, has been associated with high fat intake in the obese [7,12,32]. Hence, an agent that can restore the decreased oro-sensory capacity to detect dietary fatty acids in the obese will be a good therapeutic agent. In the present study, we observed that a HFD decreased the gustatory preference for a dietary LCFA in accordance with several reports [10,16]. Decreased sensitivity to a fatty acid might be due to the downregulation of CD36 in taste bud cells [10]. Though we did not analyze the expression of lipid sensors (CD36 and GPR120) at protein level by Western blot, our RT-qPCR analyses suggest a decreased CD36 mRNA expression in TBCs in obese mice, and the administration of Celastrol upregulated it. Concerning the mechanism of action of Celastrol on fat taste sensors, we can state that this terpenoid might have decreased the taste bud inflammation in obese mice. Indeed, the inflammation, induced either by a high-fat diet or by treatment with dextran sodium sulfate (DSS), has been reported to decrease taste bud functions in the mice [33,34]. Furthermore, there seems to be an “opposite” tendency between CD36 and GPR120 mRNA expression. Hence, we would like to state that CD36 is involved in “detection”, whereas GPR120 is responsible for the “post-oral regulation” of fat-eating behavior as we have previously proposed [10]. This kind of opposite expression of CD36 and GPR120 has been previously shown in rodents [10]. The upregulation of CD36 by Celastrol might be responsible to restore the oro-sensory capacity to detect an LCFA in the mice, as reported previously that decreased fat taste perception in obese mice can be restored by chemical compounds that decrease obesity and inflammation [35]. We do not think that there would be a direct action of Celastrol on fat taste receptor activation. In addition, decreased gustducin mRNA expression has been associated with high inflammatory conditions [36]. We were tempted to assess whether diet-induced obesity in our model was associated with taste bud inflammation. We employed the RT-qPCR technique and observed that obesity induced by a HFD was associated with upregulated inflammatory cytokines (IL-1β, IL-6 and TNF-α) mRNA, which could affect taste bud renewal and also result into low taste-detection capacity as suggested by Wang et al. [37]. In addition, our team has studied the inflammation in taste bud cells and demonstrated that diet-induced obesity or LPS-triggered inflammation increased TNF-α, IL-1β, and IL-6 expression, both at mRNA and protein levels, in taste bud cells [38]. Cohen et al. [39] have demonstrated that TNF-α and IL-6 might decrease the renewal of taste bud cells, exert an impact on the proliferation of type II cells, and, therefore, alter taste perception. We would like to recall that fat taste receptors (CD36 and GPR120) are expressed by Type II TBC in mice [10]. TNF-α in TBCs is produced via the toll-like receptor (TLR) pathway by Type II cells but not by Type I and Type III cells [40].

As regards systemic inflammation, we determined the circulating concentrations of IL-6 and TNF-α in blood. Dietary and genetic obesity promotes inflammation by increasing the concentrations of these pro-inflammatory cytokines [41]. It has been shown that adipose tissue, apart from secreting adipokines (adiponectin and leptin), also secretes pro-inflammatory cytokines, mainly TNF-α and IL-6. Regarding TNF-α, the following observations can be noted: (1) TNF-α is constitutively expressed by adipose tissue, and (2) genetically obese rodents (ob/ob mice and fa/fa Zucker rats) express TNF-α in their adipose tissue [42]. It has been demonstrated that adipose tissues can be the subject of macrophage infiltration during obesity. Indeed, macrophage infiltration into adipose tissue plays a key role in the pathogenesis and dysfunction of adipose tissue, thus contributing to obesity-induced inflammation [43]. These infiltrated cells represent a novel family of cell subtypes, which are CD9^+^ and DARC^+^ macrophages, principally localized in crown-like structures within adipose tissue during obesity [44]. Adipose tissue from obese subjects contains significantly more TNF-α than lean subjects [45,46]. The administration of the TNF-α receptor protein that binds to endogenous TNF-α normalizes insulin sensitivity [47]. It is noteworthy that between 10 and 30% of circulating IL-6 is derived from adipose tissues. There is a positive correlation between circulating IL-6 levels, adiposity [48], and insulin resistance [49]. The action of Celastrol on decreasing the circulating levels of IL-6 and TNF-α demonstrates its anti-inflammatory property that can be again beneficial in the obese. Our observations corroborate the findings of Wang et al. [50], who have demonstrated the anti-inflammatory action of this terpenoid during liver fibrosis in the mice.

Diet-induced obesity resulted into increased liver weight and triglyceride and cholesterol levels, and Celastrol exerted beneficial effects on these parameters, indicating that this terpenoid might modulate liver lipid metabolism. Furthermore, we investigated the mRNA expression profile of lipogenic (FAS, ACC1, and ACC2) and energy expenditure-related transcription factors (PPARα, SREBP1c) in the liver. FAS, ACC1, and ACC2 are lipogenic enzymes [40,41], and SREBP1c is a transcription factor induced by high glucose concentration in the liver [42]. We observed that their expression was upregulated in the liver of diet-induced obese mice in accordance with several findings that have reported an increase in the hepatic mRNA expression of SREBP1c, FAS, and SCD1 in obesity [51]. It seems that there is an association between PPAR-α and SREBP1c expression in our study. Indeed, the mRNA of SREBP1c was found downregulated in PPARα-null mice [52]. Interestingly, Celastrol treatment downregulated the expression of these lipogenic genes in obese mice. Our observations suggest that Celastrol may lower hepatic lipid accumulation via SREBP1c-mediated transcription pathway during obesity. Our results corroborate several reports that have shown that Celastrol modulates lipid metabolism; for example, it suppresses ER stress and lipogenesis and promotes hepatic lipolysis [53]. Furthermore, Celastrol has been found to effectively suppress high-fat diet-mediated increased levels of TC, TG, and LDL-c by improving ATP-binding cassette transporter A1 (ABCA1) expression [54]. In the present study, we did not investigate the impact of Celastrol on carbohydrate metabolism; however, it has been reported that this terpenoid remarkably attenuated diet-induced obesity via enhanced glucose utilization [55]. Indeed, Celastrol was found to improve insulin sensitivity and glucose tolerance in obese animals. Celastrol notably increased mitochondrial oxidative functions by increasing pyruvate dehydrogenase complex (PDC) activity and decreasing pyruvate dehydrogenase kinase 4 (PDK4) in obese mice [55].

## 5. Conclusions

We have observed anti-obesity, fat taste modulatory and anti-inflammatory actions of Celastrol in obese mice. Celastrol also normalized liver weight and hepatic cholesterol and triglyceride levels. However, our study has some limitations as we did not study the expression of pro-inflammatory cytokines and other factors at protein level by Western blot in liver and taste bud cells. In future studies, on the basis of structural properties of this terpenoid, it may be envisaged to synthesize more stable pharmacological analogs that might be more potent anti-obesity agents than the lead molecule.

## Figures and Tables

**Table 1:** Fatty acid composition of the diets.

Fatty Acids (g/100 g)	STD	HFD
SFA	0.56	15.42
MUFA	0.78	13.46
PUFA	1.62	4.75

Abbreviations: STD: standard diet. HFD: high fat-diet. SFA: saturated fatty acids. MUFA: monounsaturated acids. PUFA: polyunsaturated acids.

**Table 2:** Composition of the diets.

Composition (g/100 g)	STD	HFD
Starch	66.8	40.07
Proteins	16.10	14.6
Fats	3.10	35.3
Cholesterol	-	0.03
Cellulose	3.9	2.7
Vitamins	5.0	3.4
Minerals	5.1	3.9
Energy (Kcal 100 g)	359.5	536.65
Fat Energy (% of total Energy)	8.0	60.0

Abbreviations: STD: standard diet. HFD: high fat-diet.

**Table 3:** Sequences of the primers.

Gene	Primer Sequence
Beta-Actin	Forward: TGTTACCAACTGGGACGACA
	Reverse: CTGGGTCATCTTTTCACGGT
Gustducin	Forward: ACACATTGCAGTCCATCCTAGC
	Reverse: ATCACCATCTTCTAGTGTATTTGCC
CD36	Forward: ATGGGCTGTGATCGGAACTG
	Reverse: TTTGCCACGTCATCTGGGTTT
GPR120	Forward: GTGCCGGGACTGGTCATTGTG
	Reverse: TTGTTGGGACACTCGGATCTGG
IL-1β	Forward: CACAGCAGCACATCAACAAG
	Reverse: GTGCTCATGTCCTCATCCTG
IL-6	Forward: CCGCTATGAAGTTCCTCTCTGC
	Reverse: ATCCTCTGTGAAGTCTCCTCTCC
TNF-α	Forward: CCCTCACACTCAGATCATCTTCT
	Reverse: GCTACGACGTGGGCTACAG
PPARα	Forward: AGAGCCCCATCTGTCCTCTC
	Reverse: ACTGGTAGTCTGCAAAACCAAA
SREBP1c	Forward: CCCACCTCAAACCTGGATCT
	Reverse: AAGCAGCAAGATGTCCTCCT
FAS	Forward: GGCTCTATGGATTACCCAAGC
	Reverse: CCAGTGTTCGTTCCTCGGA
ACC1	Forward: CGGACCTTTGAAGATTTTGTGAGG
	Reverse: GCTTTATTCTGCTGGGTGAACTCTC
ACC2	Forward: GGAAGCAGGCACACATCAAGA
	Reverse: CGGGAGGAGTTCTGGAAGGA

## Data Availability

Data can be made available by the corresponding author upon rea- sonable request.
